# Supplementary material for: Simulating the effect of ankle plantarflexion and inversion-eversion exoskeleton torques on center of mass kinematics during walking
Source: PLoS Comput Biol. 2023 Aug 7;19(8):e1010712. doi: 10.1371/journal.pcbi.1010712 (PMC10434928; doi:10.1371/journal.pcbi.1010712)
Supplement: S1 Appendix — (PDF) [file pcbi.1010712.s001.pdf]

Simulating the effect of ankle plantarflexion and inversion-eversion exoskeleton torques on center of mass kinematics during walking

S1 Appendix

Nicholas A. Bianco[1*], Steven H. Collins[1], Karen Liu[2], Scott L. Delp[1,3,4]

1 Department of Mechanical Engineering, Stanford University, Stanford, California, United States of America
2 Department of Computer Science, Stanford University, Stanford, California, United States of America
3 Department of Bioengineering, Stanford University, Stanford, California, United States of America
4 Department of Orthopaedic Surgery, Stanford University, Stanford, California, United States of America

* nbianco@stanford.edu

Optimal control problem

We solved optimal control problems that tracked experimental gait data to create our simulations of normal walking. Each problem was solved using direct collocation with the OpenSim Moco software package [1]. Custom problems with multiple objectives and constraints were constructed using the *MocoStudy* interface. In the following descriptions, OpenSim and Moco class names are denoted with italics.

Cost function

The cost function consisted of five terms with individual weights that minimized control effort and tracked experimental kinematics and kinetics (Eq. 1). Each term is integrated over one gait cycle of walking with initial time t_0 and final time t_f. In the equations below, the bolded symbols represent vector quantities, the tilde symbol denotes normalized quantities, and the hat symbol denotes experimental data.

$$J = w_1 J_{effort} + w_2 J_{GRF} + w_3 J_{coord} + w_4 J_{torso} + w_5 J_{feet} \tag{1}$$

We used *MocoControlGoal* to minimize the sum of squared muscle excitations (80 muscles) and torque actuator controls (3 lumbar and 10 arm actuators), integrated over the gait cycle (Eq. 2). We divided this term by the distance traveled by the model, d, to encourage the model to walk forward.

$$J_{effort} = \frac{1}{d} \int_{t_0}^{t_f} \sum_{i}^{93} e_i^2 dt \tag{2}$$

We used *MocoContactTrackingGoal* to minimize the sum of squared errors between model and experimental ground reaction forces, integrated over the gait cycle (Eq. 3).

Both the model and experimental forces were normalized based on the peak force magnitudes of the experimental forces.

$$J_{GRF} = \int_{t_0}^{t_f} \sum_{i}^{2} ||\tilde{\mathbf{F}}_i - \hat{\tilde{\mathbf{F}}}_i||_2^2 dt \tag{3}$$

We used *MocoStateTrackingGoal* to minimize the sum of squared errors between model and experimental coordinate values, q, and speeds, u, integrated over the gait cycle (Eq. 4). The experimental coordinate values (joint angles and pelvis positions) and speeds (joint velocities and pelvis linear velocities) were the trajectories computed from the *InverseKinematicsTool* in OpenSim. The weights for individual coordinate tracking errors, β_i, were normalized by the average standard deviation in each coordinate across three gait cycles. Since ankle kinematics were important to the goals of this study, we doubled the weight applied to the ankle angle tracking errors. Since our experimental data were collected from treadmill walking, the pelvis fore-aft position was translated forward according to the experimental treadmill speed to represent overground walking. The subtalar and metatarsophalangeal joints did not track experimental data since we did not have reliable experimental kinematics for these joints. We also did not track lumbar coordinates since we directly tracked torso orientations. Finally, we did not track the vertical position of the pelvis which improved ground reaction force tracking. Therefore, out of the 31 model coordinates, we tracked 25 coordinate values and 26 coordinate speeds.

$$J_{coord} = \int_{t_0}^{t_f} \sum_{i}^{25} \beta_i (q_i - \hat{q}_i)^2 dt + 0.01 \int_{t_0}^{t_f} \sum_{i}^{26} \beta_i (u_i - \hat{u}_i)^2 dt \tag{4}$$

We used *MocoOrientationTrackingGoal* and *MocoAngularVelocityTrackingGoal* to track experimental torso and calcaneus body kinematics (Eq. 5-6). The experimental torso and calcaneus body kinematics were computed by first applying our inverse kinematics results to the model and computing body orientations and angular velocities. *MocoOrientationTrackingGoal* computes an angle-axis representation of the rotation matrix between the model and experimental body frames. Minimizing the angle, θ, from this representation minimizes the tracking error between the model and experimental body frame orientations. *MocoAngularVelocityTrackingGoal* computes the error between model and experimental three-dimensional angular velocities, $\boldsymbol{\omega}$.

$$J_{feet} = \int_{t_0}^{t_f} \sum_{i}^{2} \theta_i^2 dt + 0.01 \int_{t_0}^{t_f} \sum_{i}^{2} ||\boldsymbol{\omega}_i - \hat{\boldsymbol{\omega}}_i||_2^2 dt \tag{5}$$

$$J_{torso} = \int_{t_0}^{t_f} \theta^2 dt + 0.01 \int_{t_0}^{t_f} ||\boldsymbol{\omega} - \hat{\boldsymbol{\omega}}||_2^2 dt \tag{6}$$

The weights for each cost function term are listed in Table A1. These weights were chosen manually such that the experimental data were tracked as closely as possible while also producing good agreement between simulated muscle activity and experimental electromyography data. We also computed the contributions of each term to the total cost function value in the final tracking solutions (Table A2).

Table A1. Tracking optimization cost function weights.

weight	value
w_1	5
w_2	7500
w_3	25
w_4	10
w_5	10

Table A2. Tracking optimization cost function weights. The contributions of individual cost function terms in our tracking optimization problem to the overall cost, expressed as a percentage of the total cost function value. Results are reported as mean ± standard deviation across subjects.

cost function term	contribution to total cost (%)
J_{effort}	45.0 ± 6.7
J_{GRF}	15.7 ± 6.6
J_{coord}	20.2 ± 6.8
J_{torso}	4.6 ± 0.6
J_{feet}	14.5 ± 1.8

Problem constraints

We used *MocoPeriodicityGoal* to implement endpoint constraints so that all model states and controls were periodic across the gait cycle (i.e., initial trajectory values were equal to final trajectory values). We used *MocoFrameDistanceConstraint* to prevent the model's arms from intersecting with the torso, and to prevent the feet from intersecting with each other. Finally, we used *MocoAverageSpeedGoal* to constrain the average walking speed of the model to match the experimental walking speed.

Solver settings

We solved each problem in Moco using *MocoCasADiSolver* [2]. We used a Hermite-Simpson collocation scheme with mesh intervals at every 10 ms in the gait cycle. Each problem was solved using a constraint tolerance of 1e-4 and a convergence tolerance 1e-2 which led to good agreement between our solutions and experimental data. The tracking problems were solved using explicit dynamics for both skeletal and muscle dynamics so that the solutions could be reproduced with forward integration, which was necessary for the exoskeleton torque simulations. We used a forward difference scheme to compute function derivatives in CasADi since this reduced optimization times but did not negatively affect problem convergence. A list of important solver settings can be found in Table A3.

Center of mass acceleration and position results

Changes in center of mass acceleration and position from the muscle-driven simulations generally reflected the results we observed for the center of mass velocity changes, with some differences (S1 Fig to S5 Fig, S7 Fig). Changes in vertical center of mass positions were produced by all exoskeleton torques during late mid-stance. Similarly, changes in center of mass acceleration were produced by plantarflexion exoskeleton torque during

Table A3. *MocoCasADiSolver* settings. The solver settings used for each tracking optimization problem in OpenSim Moco. t_{cycle} represents gait cycle length which was used to compute the number of mesh intervals used for each subject.

solver setting	value
`transcription_scheme`	'hermite-simpson'
`optim_constraint_tolerance`	10^{-4}
`optim_convergence_tolerance`	10^{-2}
`num_mesh_intervals`	$\frac{t_{cycle}}{0.01s}$
`multibody_dynamics_mode`	'explicit'
`optim_finite_difference_scheme`	'forward'
`scale_variables_using_bounds`	true

mid-stance. Changes in center of mass position were observed in late mid-stance for the inversion and plantarflexion plus inversion torques. Finally, no significant changes in position changes were detected in early mid-stance from plantarflexion torque.

Changes in center of mass acceleration and position produced by the torque-driven simulations generally reflected the results we observed for the center of mass velocity changes, with some small differences in the fore-aft direction (S5 Fig and S7 Fig, diamonds; Tukey post-hoc tests, $p < 0.05$). During late mid-stance, all torque-driven simulations produced significantly larger changes in fore-aft center of mass position and acceleration. Specifically, inversion torque produced significantly larger changes in forward center of mass acceleration during mid-stance.

Muscle activity validation

We compared muscle activations to experimental electromyography (EMG) to validate our simulated muscle activity predictions. Individual RMS errors between muscle activations and EMG signals can be found in Table A4.

References

1. Dembia CL, Bianco NA, Falisse A, Hicks JL, Delp SL. OpenSim Moco: Musculoskeletal optimal control. PLoS Computational Biology. 2020;16(12):e1008493. doi:10.1371/journal.pcbi.1008493.

2. Andersson JAE, Gillis J, Horn G, Rawlings JB, Diehl M. CasADi: a software framework for nonlinear optimization and optimal control. Mathematical Programming Computation. 2019;11:1–36. doi:10.1007/s12532-018-0139-4.

3. Seth A, Pandy MG. A Neuromusculoskeletal Tracking Method for Estimating Individual Muscle Forces in Human Movement. Journal of Biomechanics. 2007;40(2):356–366. doi:10.1016/j.jbiomech.2005.12.017.

Table A4. RMS errors between muscle activations and electromyography. The RMS errors between simulated muscle activations and experimental EMG signals in the right leg. The EMG signals were delayed by 40 ms to account for the electromechanical delay in muscle force production [3]. Since muscle activation and EMG are both dimensionless quantities between 0 and 1, the RMS errors also lie between 0 and 1.

**muscle**	**RMS error**
gluteus maximus (superior)	0.06
gluteus maximus (middle)	0.06
gluteus maximus (inferior)	0.06
gluteus medius (anterior)	0.21
gluteus medius (middle)	0.09
gluteus medius (posterior)	0.08
rectus femoris	0.08
semimembranosus	0.09
semitendinosus	0.09
vastus lateralis	0.06
vastus medialis	0.06
biceps femoris long head	0.09
lateral gastrocnemius	0.13
medial gastrocnemius	0.20
soleus	0.11
tibalis anterior	0.11
