# Supplementary figures and images for: Simulating the effect of ankle plantarflexion and inversion-eversion exoskeleton torques on center of mass kinematics during walking
Source: PLoS Comput Biol. 2023 Aug 7;19(8):e1010712. doi: 10.1371/journal.pcbi.1010712 (PMC10434928; doi:10.1371/journal.pcbi.1010712)

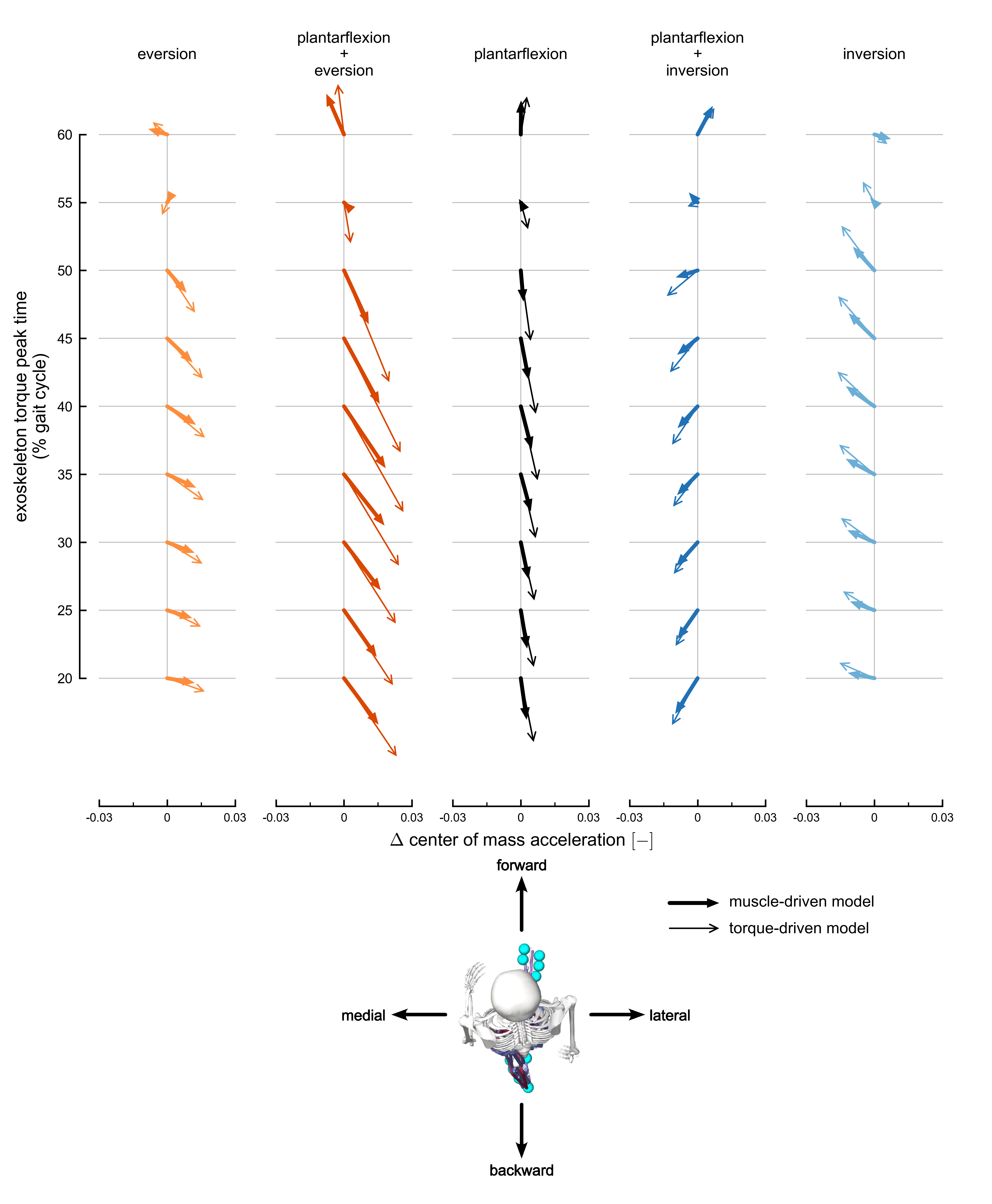

Supplement: S1 Fig — The change in center of mass acceleration, calculated at exoskeleton torque peak time, projected onto the transverse plane. The arrows represent acceleration changes normalized by gravitational acceleration and averaged across subjects. Columns represent acceleration changes for each exoskeleton torque condition: eversion (light orange), plantarflexion plus eversion (dark orange), plantarflexion (black), plantarflexion plus inversion (dark blue), and inversion (light blue). Thick arrows with filled heads represent changes using the muscle-driven model; thin arrows with open heads represent results using the torque-driven model. Each column includes acceleration changes at different exoskeleton timings, ranging from 20% (bottom) to 60% (top) of the gait cycle. The horizontal arrow directions are medio-lateral changes in acceleration, and the vertical arrow directions are fore-aft changes in acceleration. The horizontal axes provide scales for medio-lateral acceleration changes, and the fore-aft changes represented by each arrow are scaled to match the horizontal axis. The maximum transverse acceleration change observed across both muscle-driven and torque-driven conditions was 0.55 m s-2. (TIF) [file pcbi.1010712.s002.tif]

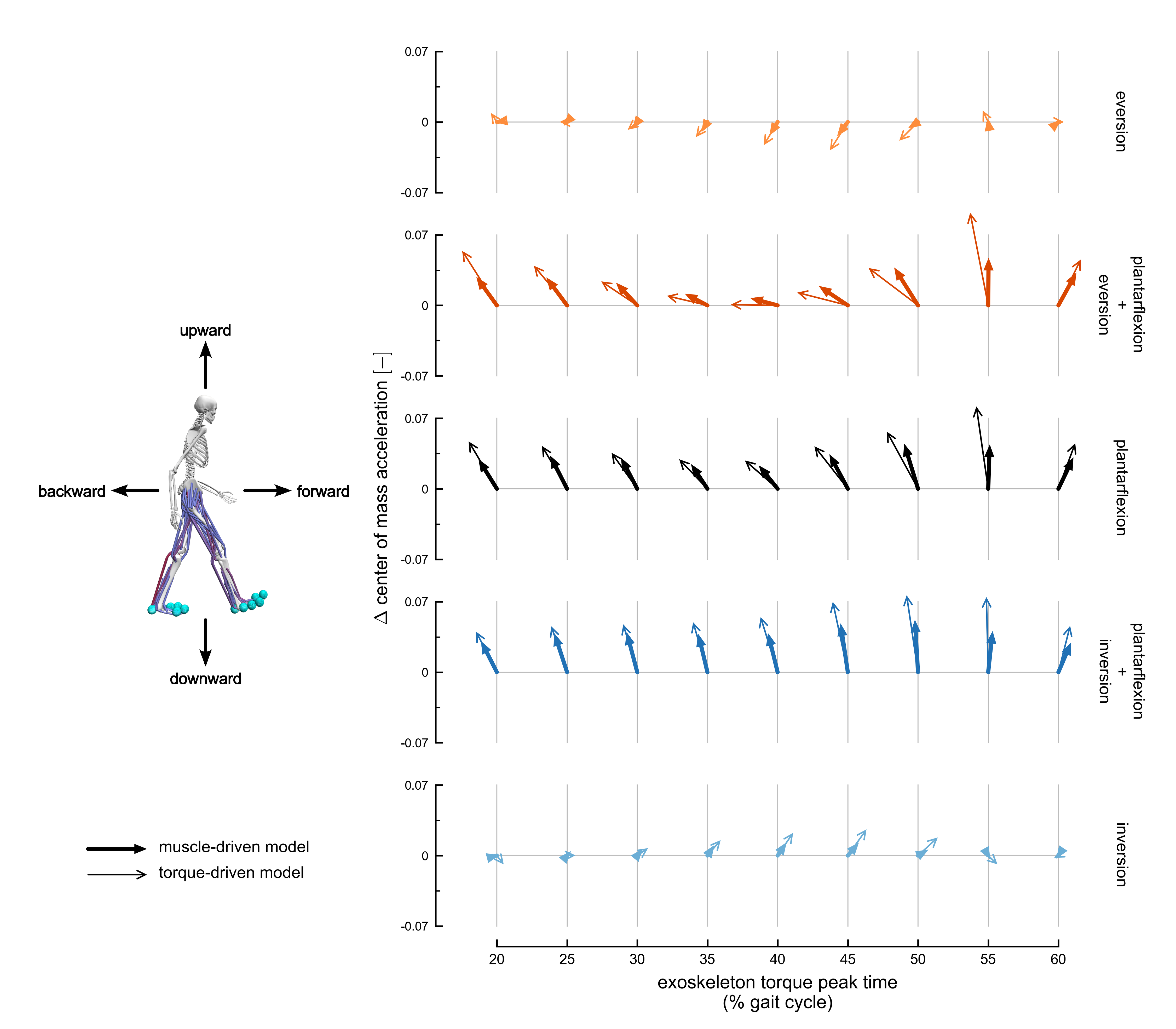

Supplement: S2 Fig — The change in center of mass acceleration, calculated at exoskeleton torque peak time, projected onto the sagittal plane. The arrows represent acceleration changes normalized by gravitational acceleration and averaged across subjects. Rows represent acceleration changes for each exoskeleton torque condition: eversion (light orange), plantarflexion plus eversion (dark orange), plantarflexion (black), plantarflexion plus inversion (dark blue), and inversion (light blue). Thick arrows with filled heads represent changes using the muscle-driven model; thin arrows with open heads represent results using the torque-driven model. Each row includes acceleration changes at different exoskeleton timings, ranging from 20% (left) to 60% (right) of the gait cycle. The horizontal arrow directions are fore-aft changes in acceleration, and the vertical arrow directions are vertical changes in acceleration. The vertical axes provide scales for vertical acceleration changes, and the fore-aft changes represented by each arrow are scaled to match the vertical axis. The maximum sagittal acceleration change observed across both muscle-driven and torque-driven conditions was 0.93 m s-2. (TIF) [file pcbi.1010712.s003.tif]

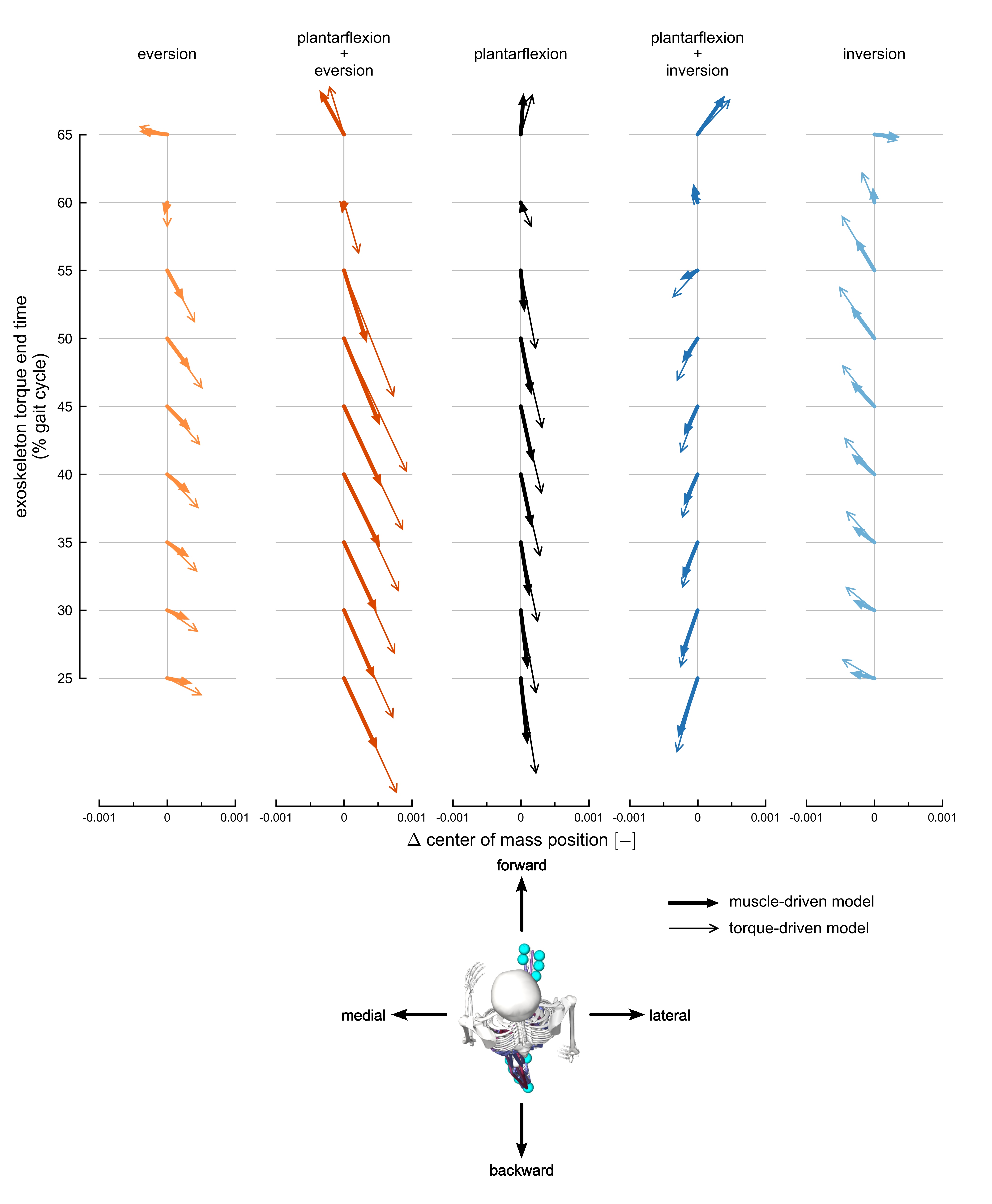

Supplement: S3 Fig — The change in center of mass position, calculated at exoskeleton torque end time, projected onto the transverse plane. The arrows represent position changes normalized by center of mass height and averaged across subjects. Columns represent position changes for each exoskeleton torque condition: eversion (light orange), plantarflexion plus eversion (dark orange), plantarflexion (black), plantarflexion plus inversion (dark blue), and inversion (light blue). Thick arrows with filled heads represent changes using the muscle-driven model; thin arrows with open heads represent results using the torque-driven model. Each column includes position changes at different exoskeleton timings, ranging from 25% (bottom) to 65% (top) of the gait cycle. The horizontal arrow directions are medio-lateral changes in position, and the vertical arrow directions are fore-aft changes in position. The horizontal axes provide scales for medio-lateral position changes, and the vertical changes represented by each arrow are scaled to match the horizontal axes. The maximum transverse position change observed across both muscle-driven and torque-driven conditions was 0.002 m. (TIF) [file pcbi.1010712.s004.tif]

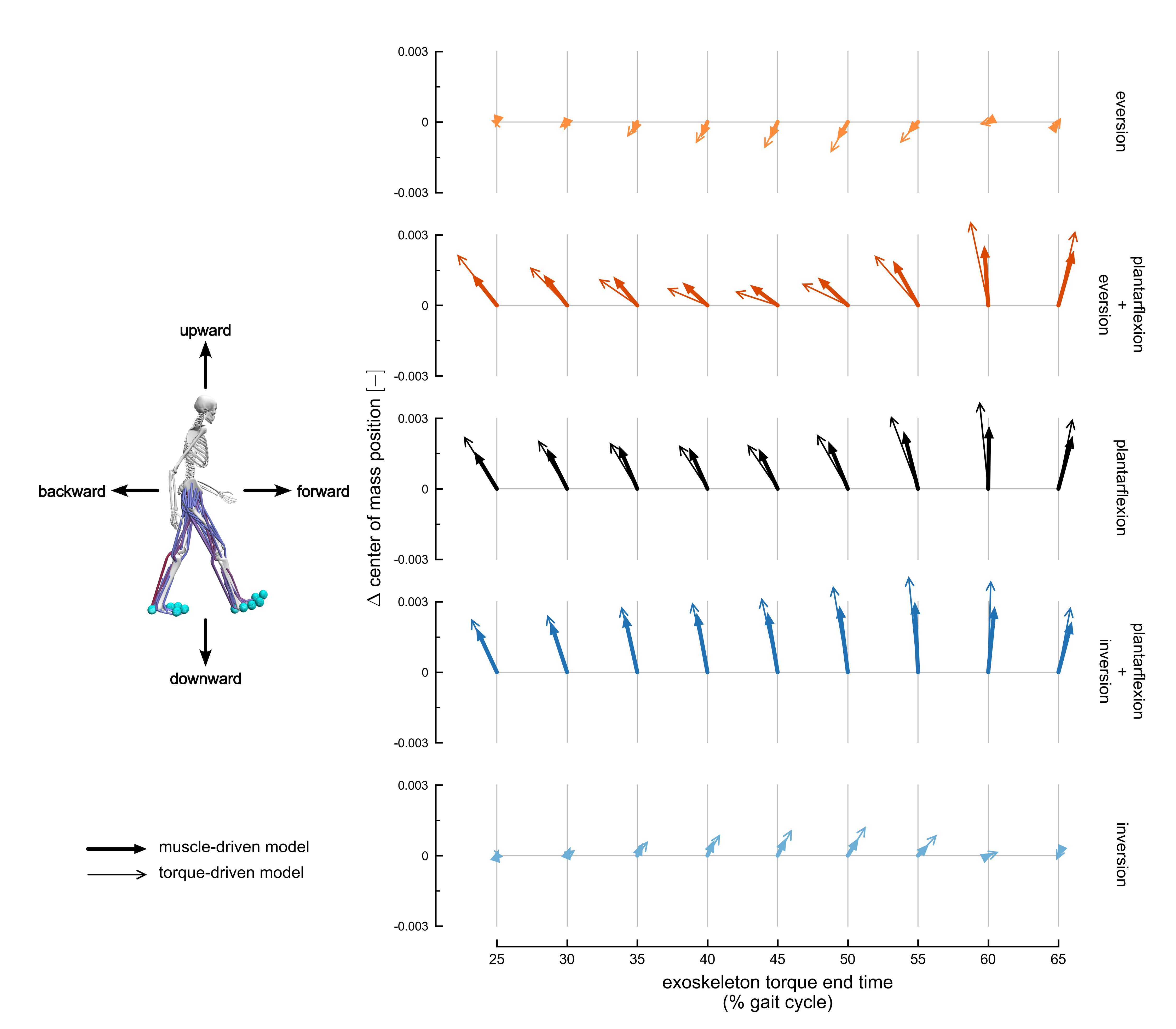

Supplement: S4 Fig — The change in center of mass position, calculated at exoskeleton torque end time, projected onto the sagittal plane. The arrows represent position changes normalized by center of mass height and averaged across subjects. Rows represent position changes for each exoskeleton torque condition: eversion (light orange), plantarflexion plus eversion (dark orange), plantarflexion (black), plantarflexion plus inversion (dark blue), and inversion (light blue). Thick arrows with filled heads represent changes using the muscle-driven model; thin arrows with open heads represent results using the torque-driven model. Each row includes position changes at different exoskeleton timings, ranging from 25% (left) to 65% (right) of the gait cycle. The horizontal arrow directions are fore-aft changes in position, and the vertical arrow directions are vertical changes in position. The vertical axes provide scales for vertical position changes, and the fore-aft changes represented by each arrow are scaled to match the vertical axis. The maximum sagittal position change observed across both muscle-driven and torque-driven conditions was 0.004 m. (TIF) [file pcbi.1010712.s005.tif]

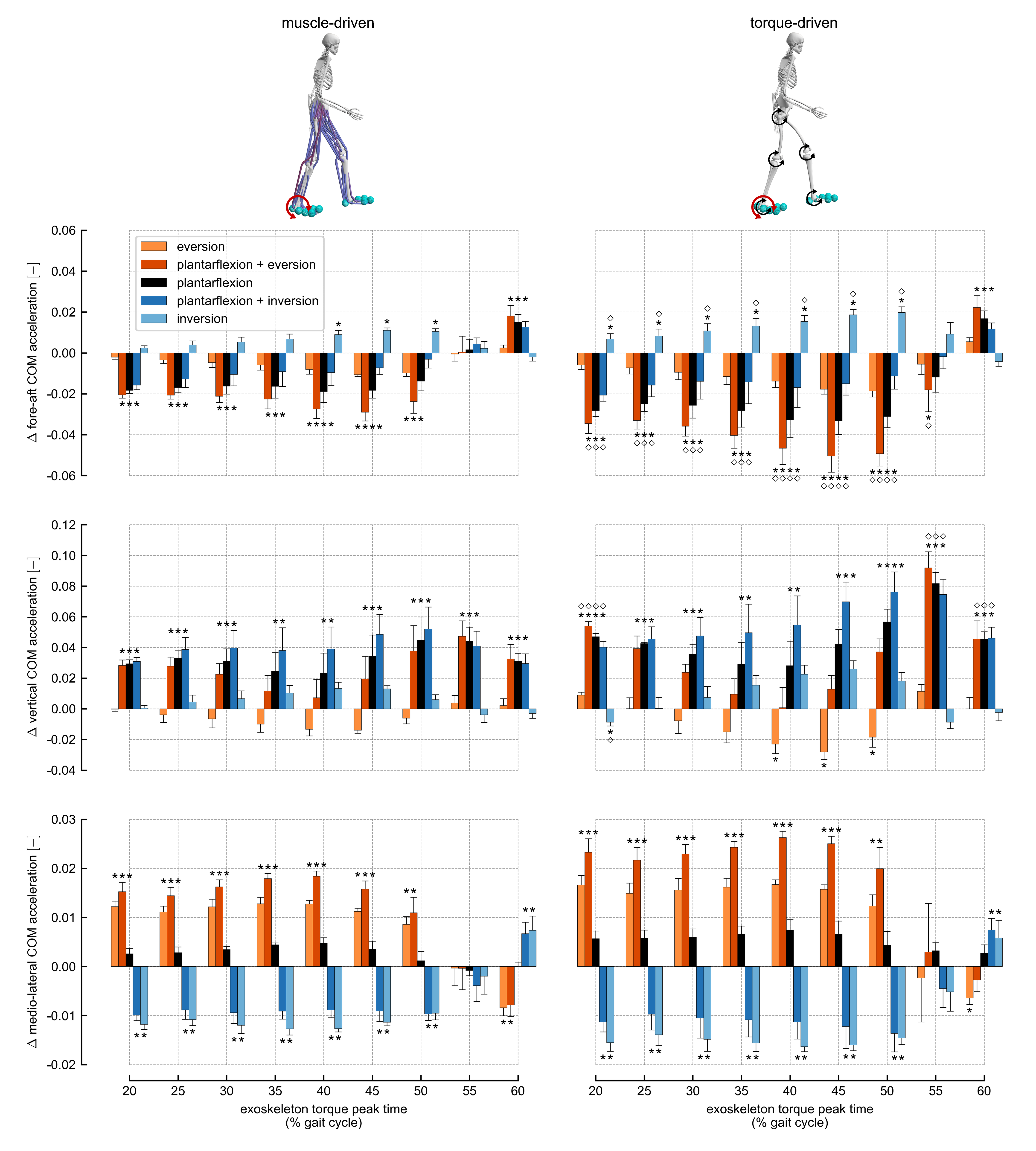

Supplement: S5 Fig — The change in center of mass acceleration, calculated at exoskeleton torque peak time, for each exoskeleton torque condition: eversion (light orange), plantarflexion plus eversion (dark orange), plantarflexion (black), plantarflexion plus inversion (dark blue), and inversion (light blue). The bars represent acceleration changes normalized by gravitational acceleration and averaged across subjects; error bars represent standard deviations across subjects. The left column represents changes using the muscle-driven models, and the right column represents changes using the torque-driven models. Asterisks above bars represent statistically significant changes relative to normal walking condition; diamonds above bars in the right column represent changes from torque-driven simulations that were statistically different from changes from muscle-driven simulations. The maximum acceleration change observed across both muscle-driven and torque-driven conditions was 0.93 m s-2. (TIF) [file pcbi.1010712.s006.tif]

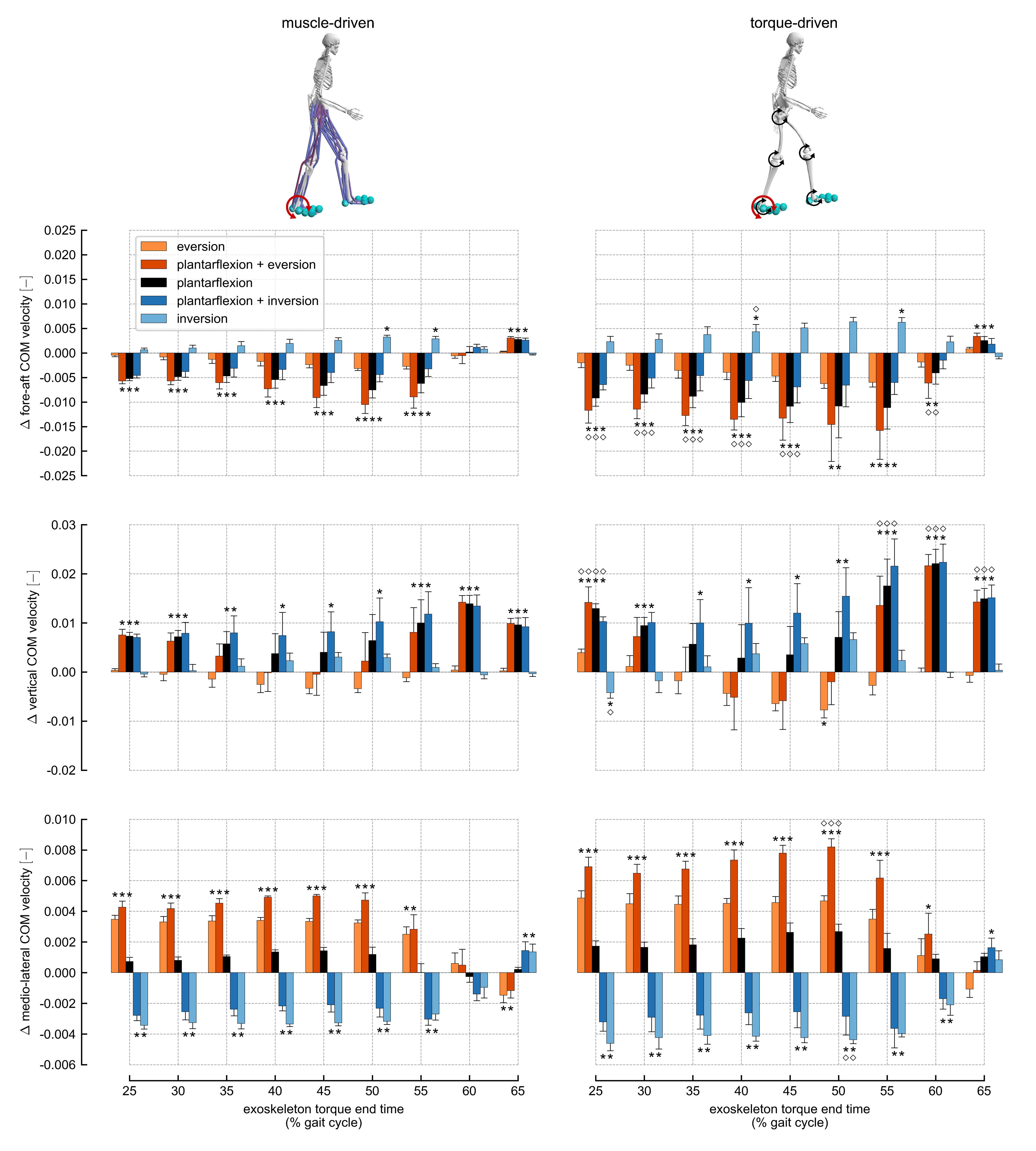

Supplement: S6 Fig — The change in center of mass velocity, calculated at exoskeleton torque end time, for each exoskeleton torque condition: eversion (light orange), plantarflexion plus eversion (dark orange), plantarflexion (black), plantarflexion plus inversion (dark blue), and inversion (light blue). Velocity changes are normalized to the dimensionless Froude number. The bars represent normalized velocity changes averaged across subjects; error bars represent standard deviations across subjects. The left column represents changes using the muscle-driven models, and the right column represents changes using the torque-driven models. Asterisks above bars represent statistically significant changes relative to normal walking condition; diamonds above bars in the right column represent changes from torque-driven simulations that were statistically different from changes from muscle-driven simulations. The maximum velocity change observed across both muscle-driven and torque-driven conditions was 0.072 m s-1. (TIF) [file pcbi.1010712.s007.tif]

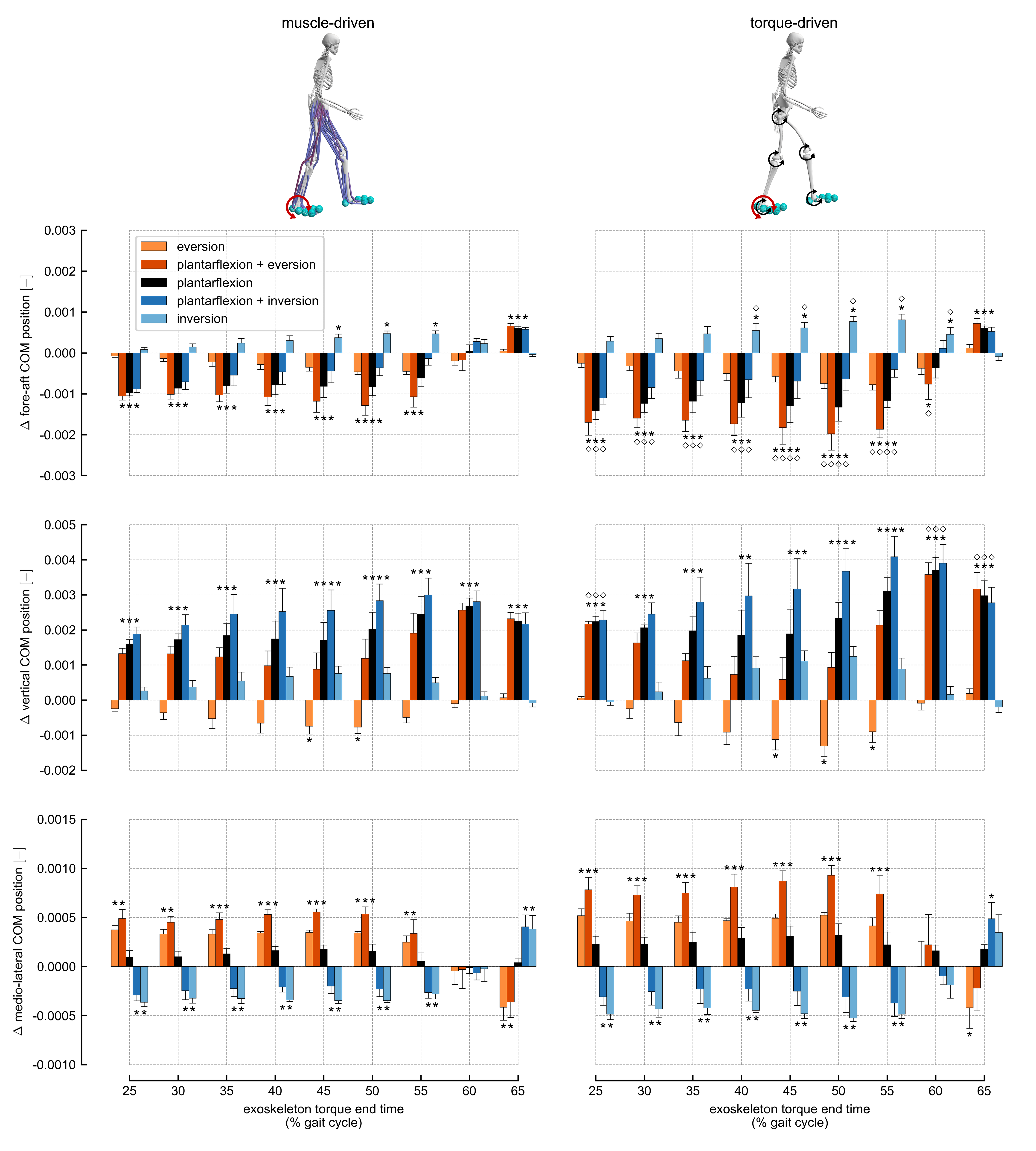

Supplement: S7 Fig — The change in center of mass position, calculated at exoskeleton torque end time, for each exoskeleton torque condition: eversion (light orange), plantarflexion plus eversion (dark orange), plantarflexion (black), plantarflexion plus inversion (dark blue), and inversion (light blue). The bars represent position changes averaged across subjects; error bars represent standard deviations across subjects. The left column represents changes using the muscle-driven models, and the right column represents changes using the torque-driven models. Asterisks above bars represent statistically significant changes relative to normal walking condition; diamonds above bars in the right column represent changes from torque-driven simulations that were statistically different from changes from muscle-driven simulations. The maximum position change observed across both muscle-driven and torque-driven conditions was 0.004 m. (TIF) [file pcbi.1010712.s008.tif]

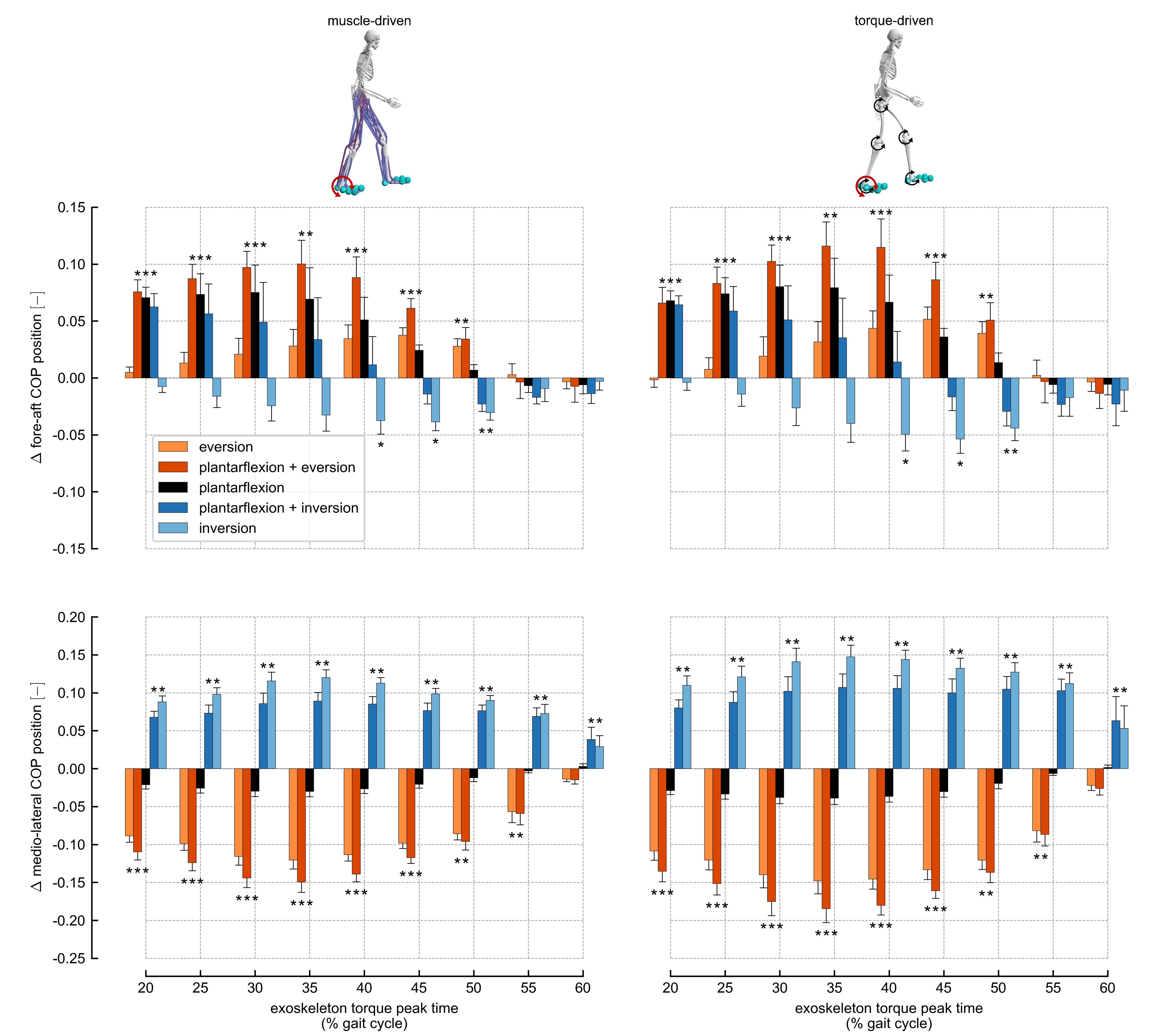

Supplement: S8 Fig — The change in right foot center of pressure position, calculated at the exoskeleton torque peak time, for each exoskeleton torque condition: eversion (light orange), plantarflexion plus eversion (dark orange), plantarflexion (black), plantarflexion plus inversion (dark blue), and inversion (light blue). The bars represent position changes averaged across subjects; error bars represent standard deviations across subjects. The left column represents changes using the muscle-driven models, and the right column represents changes using the torque-driven models. Asterisks above bars represent statistically significant changes relative to normal walking condition. No significant differences in center of pressure changes between torque-driven and muscle-driven simulations were detected. The maximum center of pressure position change observed across both muscle-driven and torque-driven conditions was 0.024 m. (TIF) [file pcbi.1010712.s009.tif]

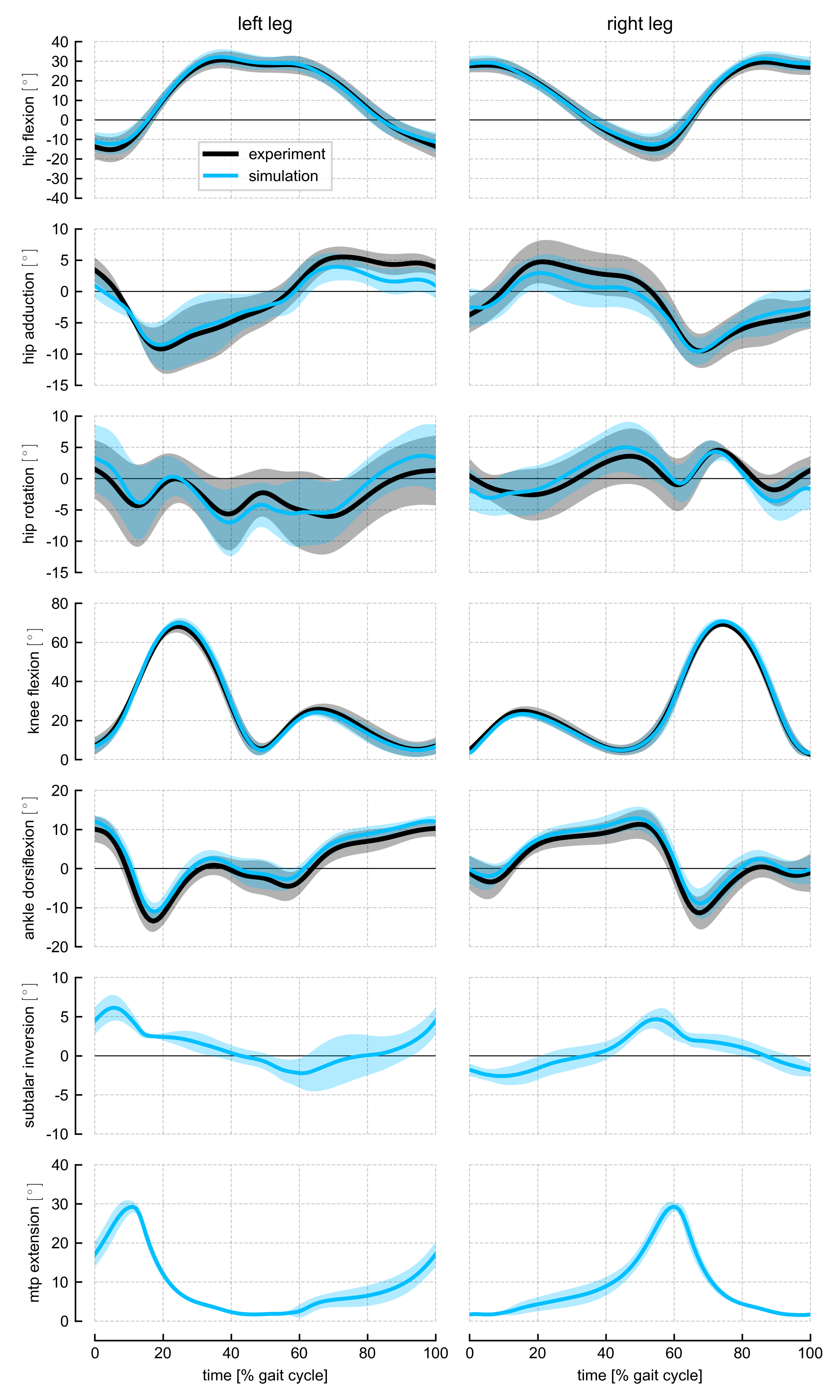

Supplement: S9 Fig — Joint angles computed from experimental data using inverse kinematics (black) compared to joint angles from the tracking simulations (blue). Solid lines represent averages across subjects, and shaded bands represent standard deviations across subjects. The subtalar and metatarsophalangeal (mtp) joints did not track any reference experimental data. (TIF) [file pcbi.1010712.s010.tif]

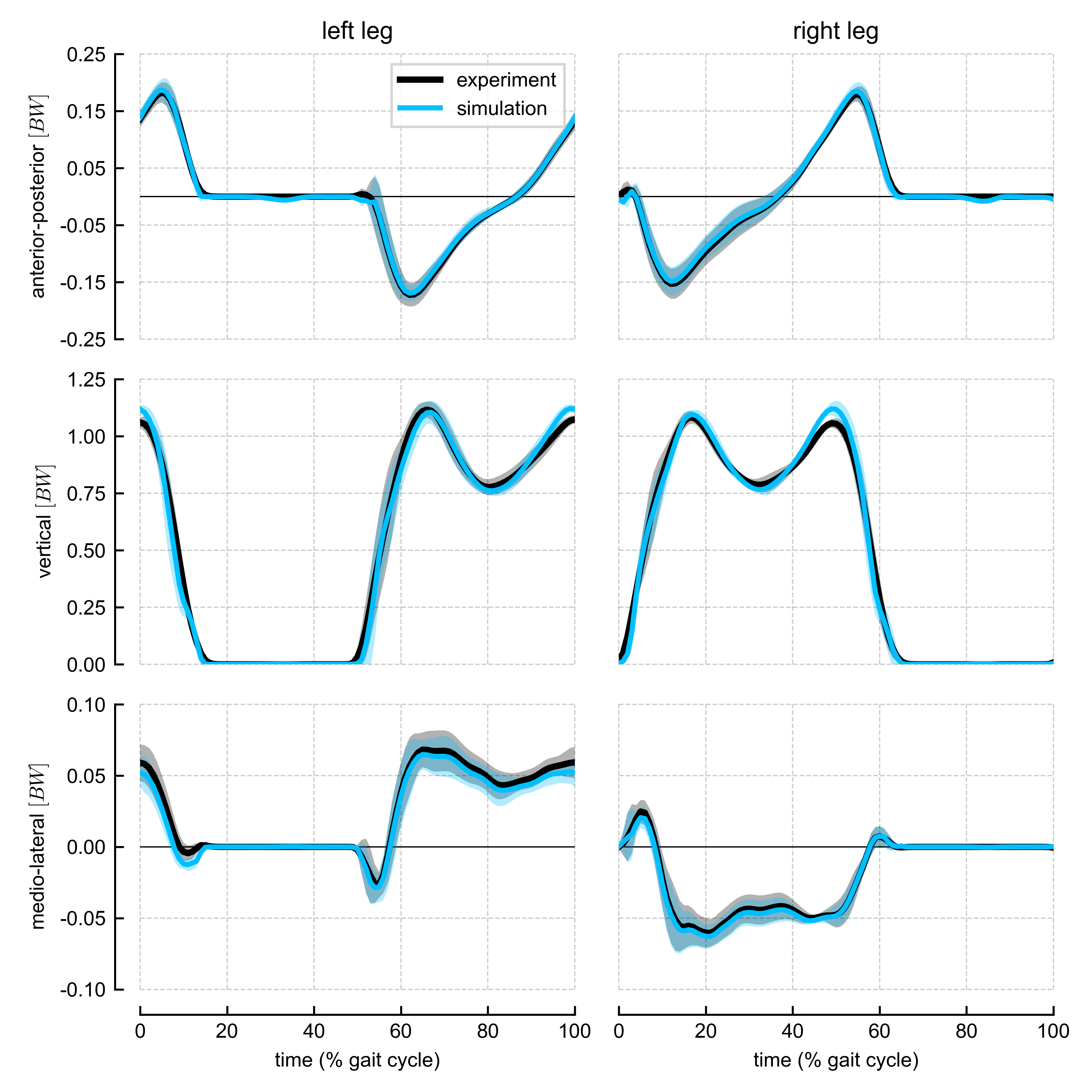

Supplement: S10 Fig — Experimental ground reaction forces (black) compared to model-generated ground reaction forces from the tracking simulations (blue). Solid lines represent averages across subjects, and shaded bands represent standard deviations across subjects. (TIF) [file pcbi.1010712.s011.tif]

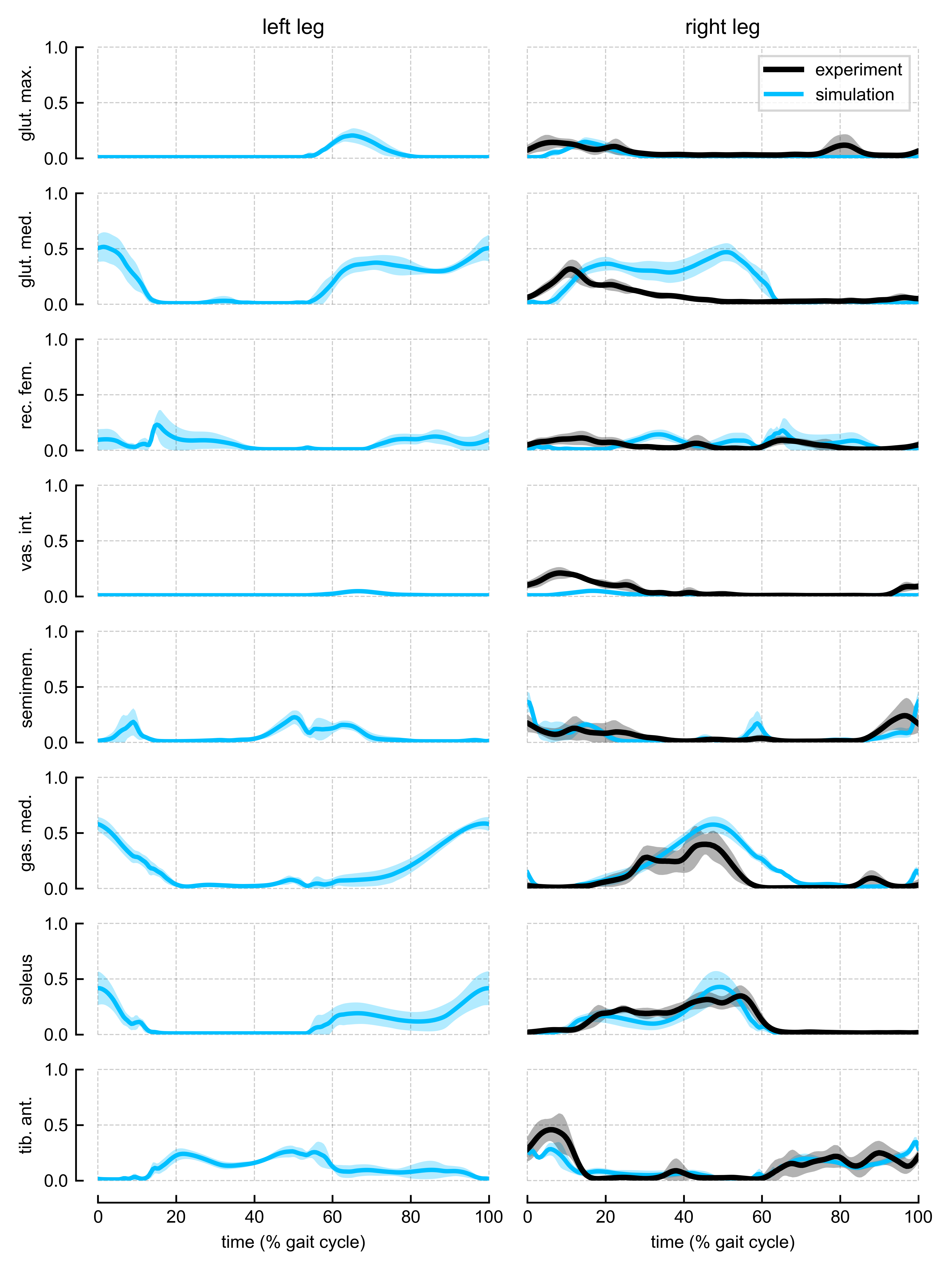

Supplement: S11 Fig — Experimental electromyography recordings (black, right leg only) compared to muscle activations from the tracking simulations (blue). To account for electromechanical delays, a 40 ms delay was applied to the experimental EMG recordings. Solid lines represent averages across subjects, and shaded bands represent standard deviations across subjects. (TIF) [file pcbi.1010712.s012.tif]

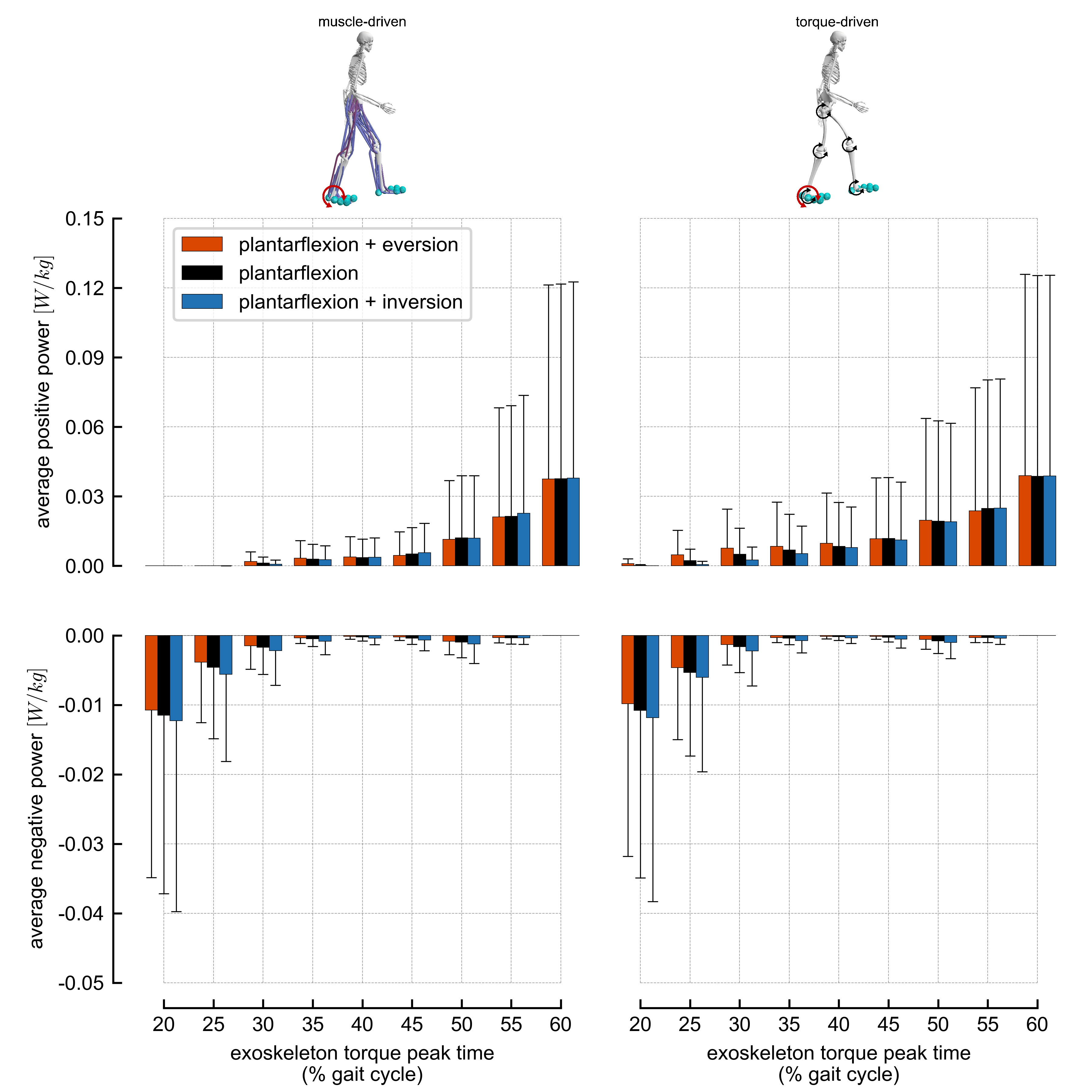

Supplement: S12 Fig — Average positive and average negative plantarflexion exoskeleton powers for the following exoskeleton torque conditions: plantarflexion plus eversion (dark orange), plantarflexion (black), plantarflexion plus inversion (dark blue). Power is averaged over the full simulation time range when exoskeleton torque was applied. Exoskeleton timings are denoted by the time of peak exoskeleton torque. For the plantarflexion plus eversion and plantarflexion plus inversion exoskeletons, only the power produced by the plantarflexion torque component is shown. Each bar represents the average positive (top) or average negative (bottom) power normalized by body mass and averaged across subjects; error bars represent standard deviations across subjects. The left column represents changes using the muscle-driven models, and the right column represents changes using the torque-driven models. Torques during push-off (60% of the gait cycle) produced the largest average positive powers, which corresponded with the largest forward center of mass velocity changes we observed. Early mid-stance torques (20% of the gait cycle) produced the largest average negative powers. Backward changes in center of mass velocity were observed throughout mid-stance, but large average negative powers did not persist beyond early mid-stance. (TIF) [file pcbi.1010712.s013.tif]

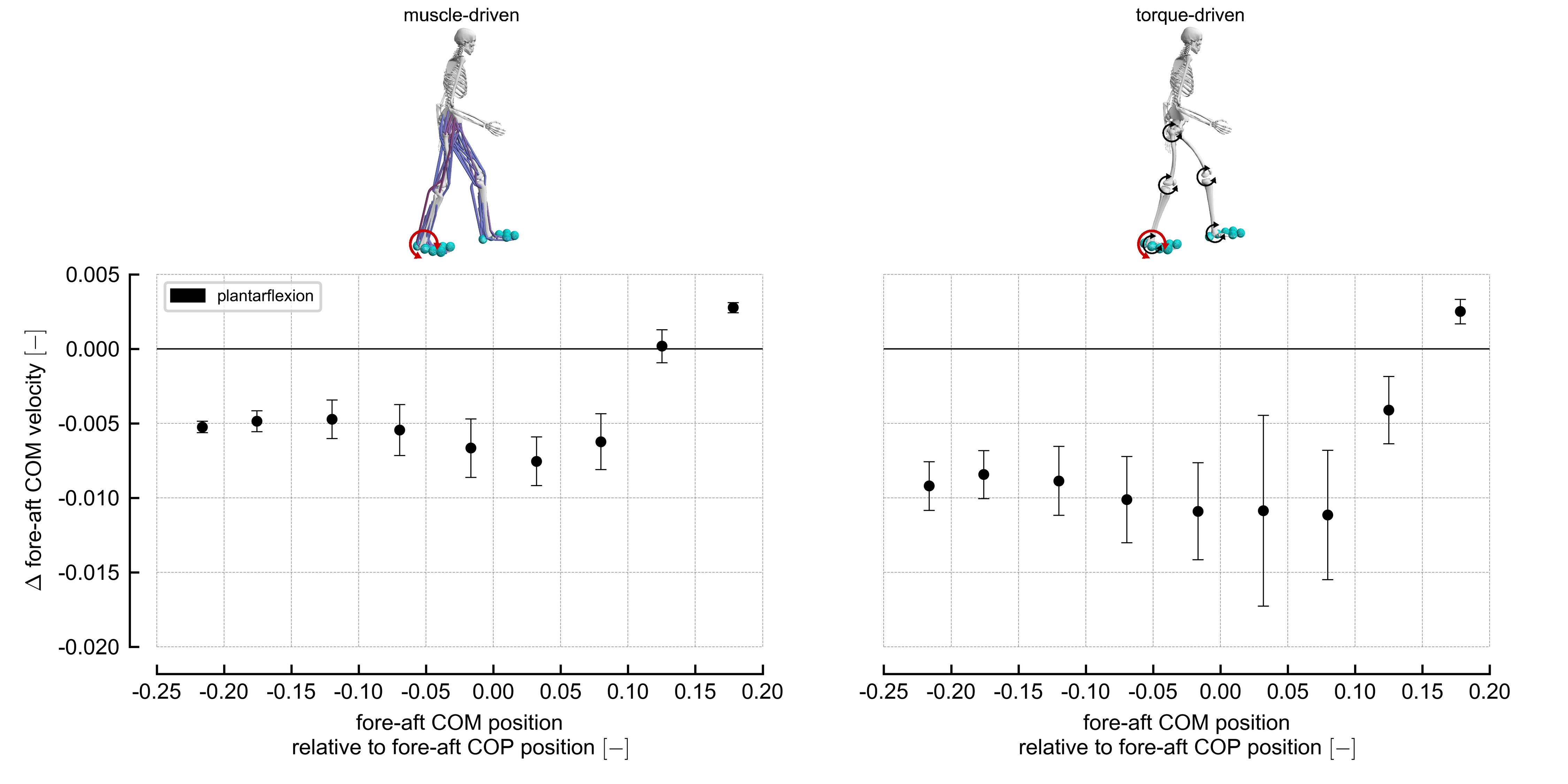

Supplement: S13 Fig — The change in fore-aft center of mass velocity, calculated at exoskeleton torque end time, for the plantarflexion exoskeleton torque condition. The x-axis represents the fore-aft center of mass position relative to the fore-aft center of pressure position, normalized by center of mass height and calculated at exoskeleton torque onset time. The filled circles represent mean values across subjects; error bars represent standard deviations across subjects. The left column represents changes using the muscle-driven models, and the right column represents changes using the torque-driven models. From left to right, the circles represent measurements from 25% to 65% of the gait cycle, corresponding to the nine exoskeleton timings we simulated. As the center of mass passed in front of the center of pressure (positive x-axis values), the velocity changes remained negative (backward) until the latest exoskeleton timings. This suggests that changes in fore-aft center of mass velocities were not strongly related to the position of the center of mass relative to the center of pressure. (TIF) [file pcbi.1010712.s014.tif]

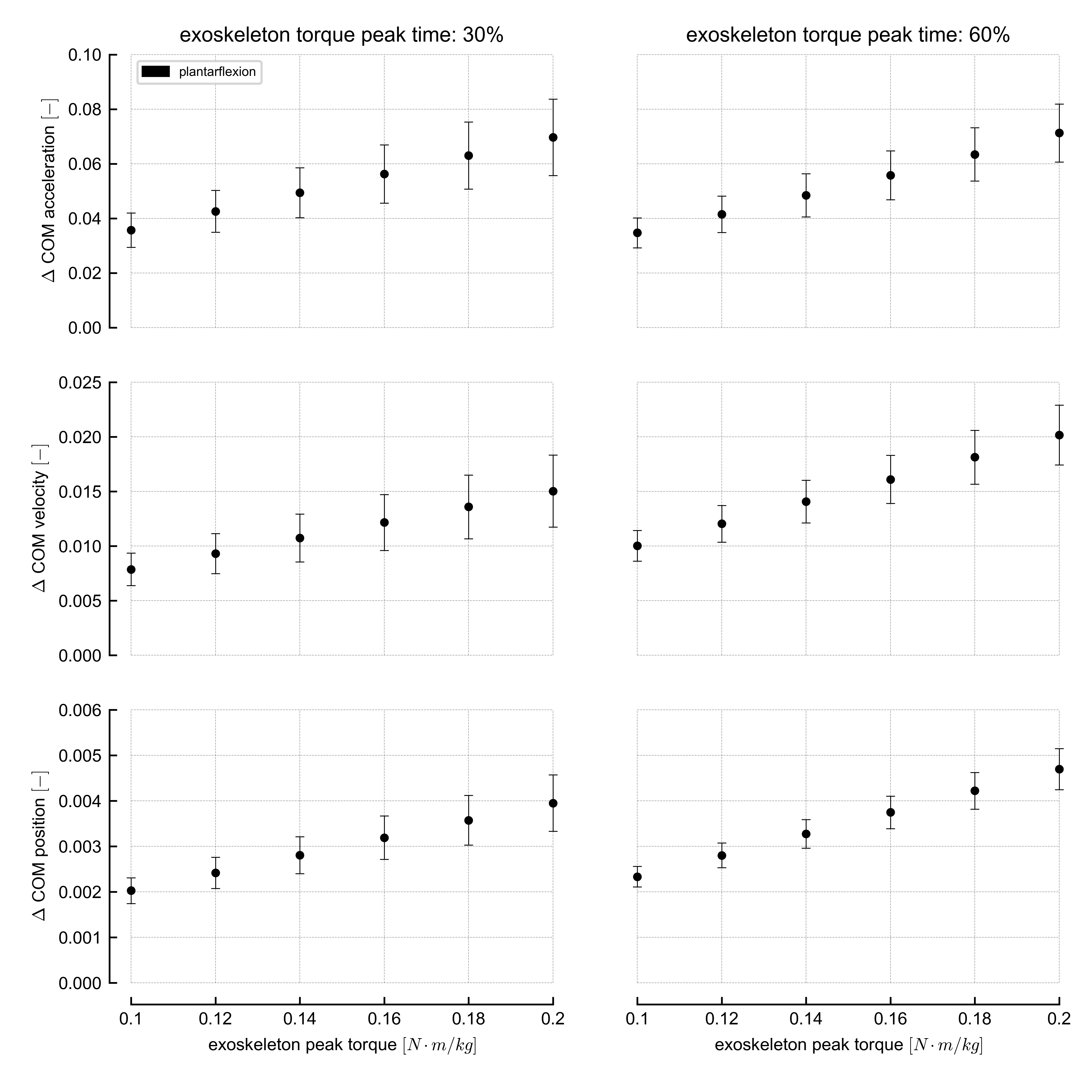

Supplement: S14 Fig — The changes in the 2-norm of the 3-dimensional center of mass position, velocity, and acceleration for the ankle plantarflexion exoskeleton torque condition versus exoskeleton peak torque. The kinematic changes were computed relative to the exoskeleton torque profile as described in Fig 1, and each quantity is normalized to be dimensionless based on the recommendations of Hof (1996) [56]. The filled circles represent mean values across subjects; error bars represent standard deviations across subjects. These results suggest that changes in these kinematic quantities have a strong linear relationship with exoskeleton peak torque. Similar linear trends hold for other exoskeleton torque conditions and timings. (TIF) [file pcbi.1010712.s015.tif]

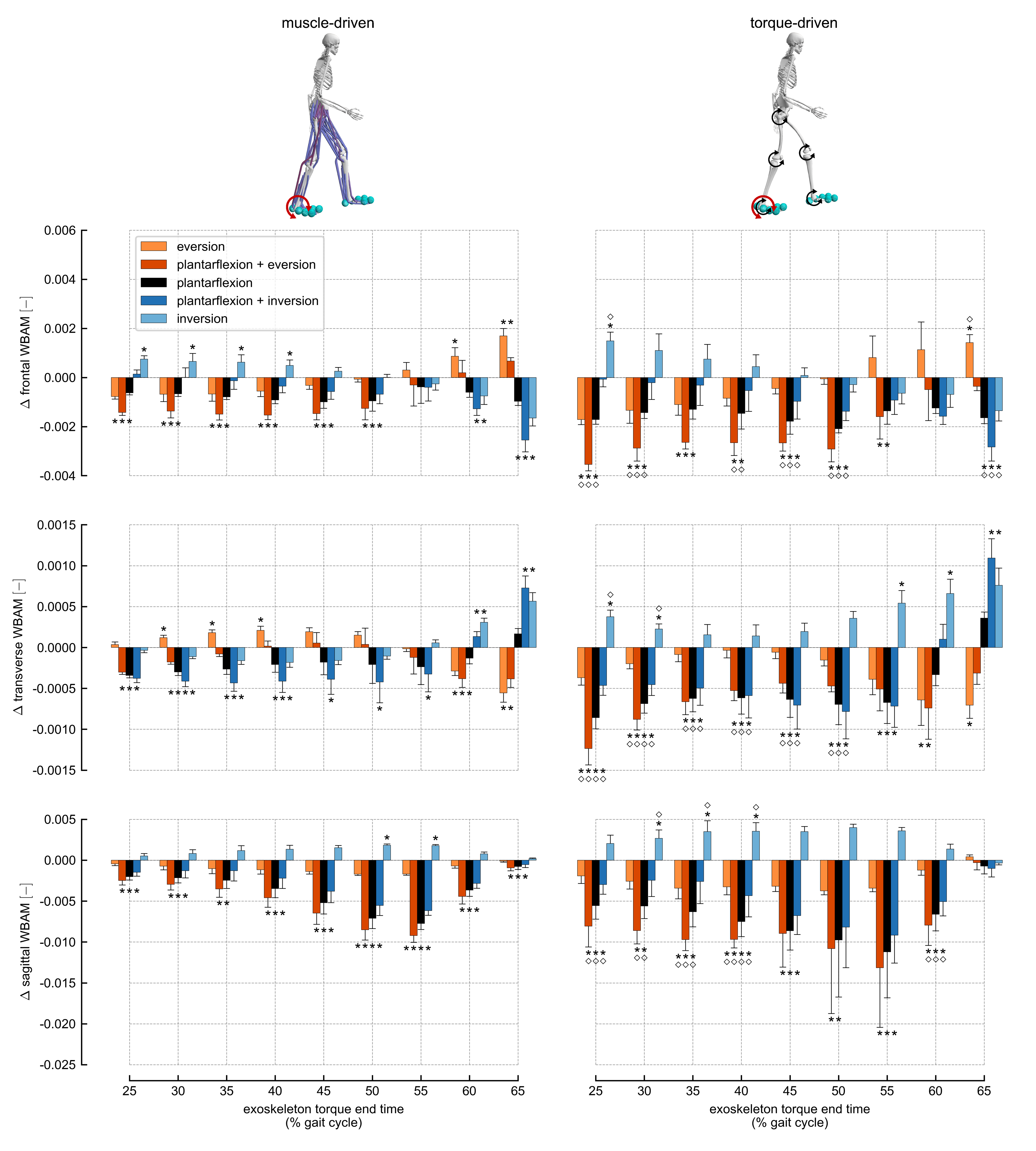

Supplement: S15 Fig — The change in whole-body angular momentum (WBAM), calculated at exoskeleton torque end time, for each exoskeleton torque condition: eversion (light orange), plantarflexion plus eversion (dark orange), plantarflexion (black), plantarflexion plus inversion (dark blue), and inversion (light blue). WBAM was normalized by mass, center of mass height, and g*hCOM [76, 77]. The bars represent WBAM changes averaged across subjects; error bars represent standard deviations across subjects. The left column represents changes using the muscle-driven models, and the right column represents changes using the torque-driven models. Asterisks above bars represent statistically significant changes relative to normal walking condition; diamonds above bars in the right column represent changes from torque-driven simulations that were statistically different from changes from muscle-driven simulations. The maximum WBAM change observed across both muscle-driven and torque-driven conditions was 3.25 kg m2 s-1. (TIF) [file pcbi.1010712.s016.tif]
